# Suicidal tendencies and attitude towards freedom to choose suicide among Lithuanian schoolchildren: results from three cross-sectional studies in 1994, 1998, and 2002

**DOI:** 10.1186/1471-2458-5-83

**Published:** 2005-08-11

**Authors:** Nida Zemaitiene, Apolinaras Zaborskis

**Affiliations:** 1 Institute for Biomedical Research, Kaunas University of Medicine, 4, Eiveniu str., Kaunas, LT-50009, Lithuania

## Abstract

**Background:**

Suicidal behaviour is increasingly becoming a phenomenon associated with young people and an important public health issue in Lithuania. However, there are very few studies evaluating impact of young peoples' attitudes towards suicide to their suicidal behaviour. A better understanding of the relations among the variables associated with suicidal ideation and threats in the normal population of adolescents may eventually result in a better understanding of the more serious forms of adolescent suicidal behaviour. The aim of the present study was to evaluate prevalence of suicidal tendencies among Lithuanian schoolchildren and to estimate its association with an attitude towards suicide in 1994 – 2002.

**Methods:**

Three country representative samples of schoolchildren, aged 11, 13 and 15, were surveyed in 1994 (n = 5428), 1998 (n = 4513), and 2002 (n = 5645) anonymously in conformity with the methodology of the World Health Organization Cross – National study on Health Behaviour in School-aged Children (HBSC).

**Results:**

About one third of respondents reported about suicidal ideation, plans or attempts to commit suicide. In the study period of eight years, the percentage of adolescents who reported sometime suicidal ideation decreased but the percentage of adolescents who declared serious suicidal behaviour remained on the same high level (8.1%, 9.8% and 8.4% correspondingly in 1994, 1998 and 2002). Moreover, the number of suicidal attempts changed from 1.0% in 1994 to 1.8% in the year 1998 and to 1,7% in the year 2002. The schoolchildren's attitude towards suicide became more agreeable: 36.6%, 41.9% and 62.5% of respondents, correspondingly in 1994, 1998 and 2002, answered that they agree with a person's freedom to make a choice between life and suicide. A multiple logistic regression analysis with low level of suicidality and high level of suicidality versus non suicidal behaviour as dependent variables for gender, age, year of the survey and attitude towards freedom to choose suicide as independent variables approved a significant association between studied covariates over the entire study period.

**Conclusion:**

Suicidal tendencies are quite frequent among Lithuanian adolescents. An increasing number of schoolchildren are expressing an agreeable attitude towards suicide. The approving attitude towards suicide among adolescents correlates with suicidal ideation and behaviour.

## Background

Suicidal behaviour is becoming a phenomenon increasingly associated with young people. The rise in the overall suicide rates in many countries is, to a large extent, due to the increase in suicides in the younger age groups. Lithuania has been among the countries with the highest suicide rate for more than ten years. It's extremely disturbing that this problem is becoming more and more associated with the youngest inhabitants of the country. Over the recent period of ten years, the mortality due to suicide in the youngest age group from birth to 19 year old has increased more than 55.8%, therefore, suicide took the third place among external causes of death [1]. According to the statistical data of the year 2001, suicide mortality in this age group was 5.85 per 100 000 of population. Boys were committing suicides more frequently than girls (10.3 versus 1.29 per 100000 of population) [2]. In comparison to the data of other countries Lithuania is loosing a great amount of young lives. Even in the neighbouring countries the incidence rate of young people's suicides is less [3].

It is important to note, that our investigation was conducted in the period between 1990 and 1996 when suicide mortality in Lithuania rose 82.4%, with the rate peaking at more than 47 per 100,000 persons in 1996. After a slight decrease in 1997 (to 45.6) and in 1998 (to 43.8), suicide rates stabilized at a very high level (in 1998–2002 the average rate was 44.6) [4].

The existing data of the previously carried out surveys in Lithuania hardly show all the extent of the phenomenon, especially while talking about children and adolescents: a lack of data do not allow estimating a degree of spread, intensity and dynamics of suicidality among young people. Furthermore, suicide is a culturally sensitive phenomenon, that is why, in order to understand it, it is necessary to begin with attitudes. Some authors conceptualise suicide as a struggle among various conflicting attitudes towards life and death [5]. As it is stated in the literature, permissive attitudes are mediating processes regarding suicide acts, therefore this issue is important in assessing risk for suicide [6-9]. In Lithuania, though, this topic has not received much attention yet, and it is still little known about young people's attitudes towards suicide. Therefore, a better understanding of the relations among the variables associated with suicidal ideation and threats in the normal population of adolescents will eventually result in a better understanding of the more serious forms of adolescent suicidal behaviour.

In this article we aimed to assess the prevalence of suicidal ideation and behaviour among Lithuanian adolescents, and its changes in the period from 1994 till 2002. Likewise we set a task to evaluate variations in attitude towards suicide and its associations with suicidal tendencies among schoolchildren.

## Methods

The study was based on the data of three surveys conducted in Lithuania in 1994, 1998 and 2002 by the methods of the WHO Cross – National study on Health Behaviour in School-aged Children (HBSC). The philosophy and methods of the HBSC project have been described in more details elsewhere [10,11].

### Participants and study procedures

The samples were expected to represent the whole country from the point of view of age, sex, nationality and the place of living. The guidelines for the survey state that at least 1500 respondents in each of three age groups – 11, 13 and 15 years – should be targeted. As the survey was planned in spring months, the appropriate grade levels corresponding to the desired age ranges were 5, 7 and 9. A stratified cluster sampling design was used to draw a representative sample of schoolchildren from the whole Lithuania. There were five strata by regions of the country (including cities Vilnius, Kaunas, Klaipeda, Siauliai and Panevezys) and three strata by language (Lithuanian, Russian and Polish) used for education at school. On the first level of sampling the schools were randomly selected from each stratum. The number of the selected schools was proportional to the size of stratum. Then 5^th^, 7^th ^and 9^th ^grades were included into the sample. If two or more classes of the desired grade level occurred in the selected school only one class was randomly selected. Afterwards we surveyed all the pupils of the selected class.

The surveys were coordinated by the laboratory for Social Pediatrics of Institute for Biomedical Research, Kaunas University of Medicine. The ethical clearance for the study and the support with relevant information were obtained from the Ministry of Education and Science as well as from the regional departments of education and the administrations of the selected schools. The study was exempt from the need for Institutional Review Board approval.

The teachers were asked to administrate the questioning and to follow the agreed guidelines. The survey was conducted in the school classes with a teacher overseeing the process. The pupils responded anonymously. We ensured the self-dependent work of the pupils and confidentiality of their answers. The completed questionnaires were enclosed into envelopes and returned to the research institution for completion. Altogether 5688, 4655 and 5761 questionnaires were returned correspondingly in 1994, 1998 and 2002. Regarding the actual number of the pupils in the lists of the selected classes the response rate for all surveys was approximately 96 percent.

The national data files were prepared and exported to the HBSC international databank at the University of Bergen (Norway). The data were checked and cleaned according to strict criteria, e.g. 90 percent of the respondents should fall within one-half a year of the mean age and the remaining 10 percent no more than one-half a year beyond this point. The schoolchildren outside the targeted age ranges were removed.

The final population of the cleaned data consisted of 5428, 4513 and 5645 schoolchildren correspondingly for the surveys in 1994, 1998 and 2002. The studied population was representative to the population of school-aged children from the whole Lithuania in respect of demographic and social values (Table 1). The groups were also well balanced according to the living environment of the respondents (urban and rural areas) and the language used for education at school (Lithuanian, Russian and Polish).

**Table 1:** Studied population in the years 1994, 1998 and 2002, by gender and age group

Years of the survey		Gender		Age group			Total
			
		Boys	Girls	11-year-olds	13-year-olds	15-year-olds	
1994	N	2 429	2 999	1 783	1 886	1 759	5 428
	%	44.8	55.2	32.9	34,7	32,4	100.0

1998	N	2 150	2 363	1 566	1 512	1 435	4 513
	%	47.6	52.4	34.7	33.5	31.8	100.0

2002	N	2 887	2 758	1 867	1 873	1 905	5 645
	%	51.1	48.9	33.1	33.2	33.7	100.0

Total	N	7 466	8 120	5 216	5 271	5 099	15 586
	%	47.9	52.1	33.5	33.8	32.7	100.0

### Measures

The survey instrument was a standardized anonymous questionnaire that included structured questions followed by alternative answers. The questionnaire topics for each survey were devised through a cooperative research among the members of the HBSC research network and finally approved by the Protocols [12].

In addition to the international standard questionnaire each country was allowed to produce a set of 'national' items for their topic area to examine the research questions more thoroughly or to explore new aspects of the topic and, thus, increase the scope of the research. Due to this agreement a focus question group concerning suicides was included into the Lithuanian version of the HBSC questionnaire.

In 1994, 1998 and 2002 surveys two questions on suicide were maintained:

1. "What do you think, does a person have the right to choose: to live or to take away his own life?" Two alternative answers were given: *'yes' *and *'no'*.

2. "Have you ever thought about suicide?" There were five alternative answers (categories) to this question: 1 = *'have never thought about that'*; 2 = *'sometimes have had such thoughts'*; 3 = *'have frequently thought about suicide'*; 4 = *'have thought about suicide seriously, even made plans how to carry it out'*; 5 = *'tried to commit suicide'*.

In order to minimize a possible impact of translation errors on the data all the versions of the questionnaires that were used in Lithuania were retranslated into English. They were approved by the Coordinating Committee of an international network of research teams.

In the analyses, the respondents were subdivided into the groups according to the chosen answer to the second question:

Group 1 – 'Non suicidal' (with no suicidal tendencies expressed). To this group were assigned those adolescents who had chosen the first variant of the possible answers *'I have never thought about suicide'*.

Group 2 – 'Low level of suicidality'. The second group was formed of the respondents who had chosen the answer *'Sometimes have had thoughts about suicide'.*

Group 3 – 'High level of suicidality'. The third group was made up of the respondents who had reported often thoughts, concrete plans and actions trying to commit suicide. Different studies prove that such manifestations show a high risk of suicide [13-15].

### Statistical analysis

Prevalence (%) of the answers to the questions on suicide and its 95% confidence intervals (CI) were calculated, stratified by sex, age and year of the study. Chi-square (χ^2^) tests were performed in order to test the sex, age and study year differences in prevalence for each of the variables. Crude odds ratios and 95% intervals (OR, 95% CI) were calculated in order to analyse associations between suicidal tendencies and attitude towards suicide in the tables 2 × 2. A multivariate analysis was performed using a logistic regression model to investigate the potential importance of adolescents' attitude towards suicide and possible confounders on dependent variables *low level of suicidality *and *high level of suicidality *versus *non suicidal*. All possible interaction terms among adolescents' attitude based on gender, age and year of the study were included and tested. The statistical analysis was performed using the SPSS software package.

## Results

### Prevalence rate of suicidal ideation and behaviour

On the whole, the question "Have you ever thought about suicide?" was answered by 15 414 (98.9%) of the respondents. The distribution of answers to this question is presented in the Table 2. It seems that suicidal tendencies were quite frequent among adolescents in general. In the period from 1994 to 1998 the variation in prevalence rates of suicidal tendencies (suicidal ideation, serious plans how to commit suicide and reported attempts to commit suicide) among the Lithuanian schoolchildren was not significant. In the period of the last four years this rate slightly decreased from 40.7% (95% CI 38.2 – 42.1) in the year 1998 to 32.5% (95% CI 31.3 – 33.8) in the year 2002 (p < 0.001). The decrease was achieved only due to decrease in the prevalence rate of 'low level of suicidality'.

**Table 2:** Frequency of answers to the question "Have you ever thought about suicide?" in the years 1994, 1998 and 2002

Level of suicidality	Category of the answer	1994		1998		2002		Total	
		
		N	%	N	%	N	%	N	%
Non suicidal	*'Have never thought about that'*	3269	60.4	2660	59.3	3722	67.5	9651	62.6
Low level of suicidality	*'Sometimes have had such thoughts'*	1706	31.5	1387	30.9	1332	24.1	4425	28.7
High level of suicidality	*'Have frequently thought about suicide'*	224	4.2	203	4.5	218	4.0	645	4.2
	*'I have thought about suicide seriously, even made plans how to carry it out'*	164	3.0	154	3.5	150	2.7	468	3.0
	*'Tried to commit suicide'*	50	0.9	79	1.8	96	1.7	225	1.5
Total		5413	100.0	4483	100.0	5518	100.0	15414	100.0

Test for homogeneity of the prevalence of answers in 1994, 1998 and 2002: χ^2 ^= 118.0; df = 8; p < 0.001.

During the study period of eight years, the prevalence rate of 'high level of suicidality' remained approximately on the same high level: 8.1%, 9.8% and 8.4% correspondingly in 1994, 1998 and 2002.

**Figure 1:**
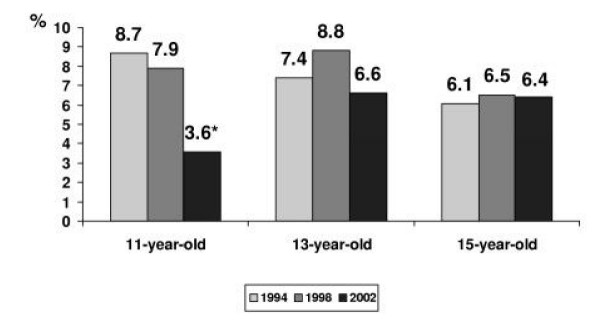
**The rate of high level of suicidality among boys**. * – p < 0.05 in comparison with the previous year of the survey.

A comparison of indicators for suicide in the groups by age and gender was made. Suicidality, in general, was increasing with age. The girls were more likely to express the ideas of suicide than the boys. According to the data received, the problem of suicidality becomes evident already in a young age. The surveys conducted in 1994 and 1998 showed that the rate of high level of suicidality was more characteristic with younger boys. The survey of the year 2002 as compared to the data of the year 1998 demonstrated a significant decrease in the prevalence of a high level of suicidality among the 11-year-old boys while the same indicators among the 15-year-old boys remained at the same level (Figure 1).

**Figure 2:**
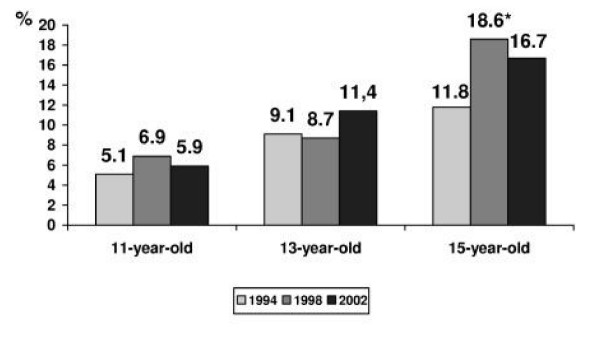
**The rate of high level of suicidality among girls**. * – p < 0.05 in comparison with the previous year of the survey.

According to the data of the surveys of the years 1994, 1998 and 2002 among the girls there was a remarkable difference in prevalence rate of high level of suicidality among age groups. The lowest prevalence of a high level of suicidality was established among the 11-year-olds and the highest prevalence of a high level of suicidality was detected among the 15-year-old girls (Figure 2).

### Attitude towards suicide as a human right

**Figure 3:**
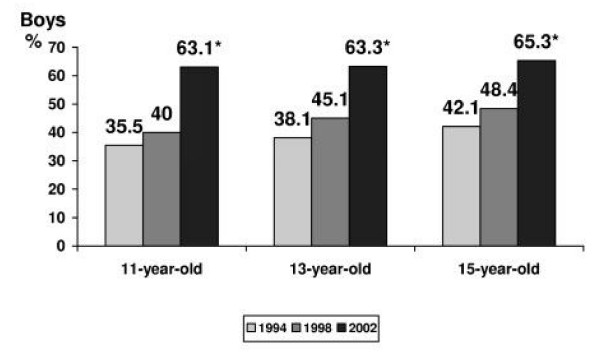
**The rate of approval attitude towards suicide among boys, by the age and year of the survey**. * – p < 0.05 by the year of the survey.

**Figure 4:**
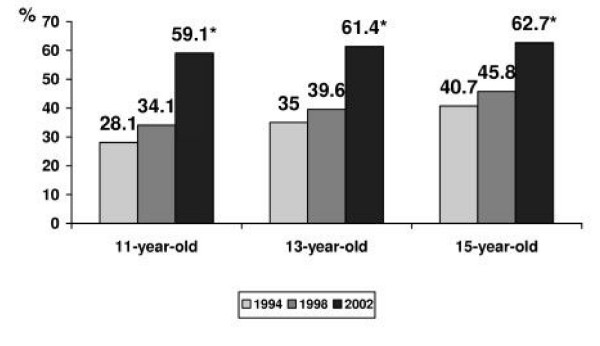
**The rate of approval attitude towards suicide among girls, by the age and year of the survey**. * – p < 0.05 by the year of the survey.

An answer to the question "What do you think, does a person have the right to choose: to live or to take away his own life?" was considered to show an affirmative or negative attitude towards suicide. The data of the surveys demonstrated that an increasing number of the Lithuanian schoolchildren were advocating suicide as a basic human right. In 1994 and 1998, correspondingly 36.3% (95% CI 35.3 – 37.9) and 41.9% (95% CI 40.5 – 43.4) (p < 0.05) of the adolescents pointed that they agreed with the freedom to make a choice between life and suicide. In 2002, an approving attitude towards suicide was declared already by 62.5% (95% CI 61.2 – 63.8) of the respondents. Gender differences were relatively small, but boys were more prone to express acceptance of the right for such a choice (in 1994 38.6% of boys versus 34.5% of girls, p < 0.05; in 1998 – 44.1% versus 39.9, p < 0.05; in 2002 – 63.9% of boys versus 61.1% of girls, p < 0.05). The Figures 3 and 4 present the variations in prevalence rate of an affirmative attitude towards suicide among boys and girls by the age and the year of the survey.

**Figure 5:**
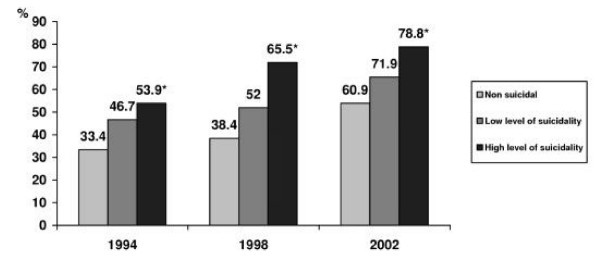
**The percentage of boys who approved a person's freedom to choose suicide, by level of suicidality in 1994, 1998 and 2002**. * – p < 0.05 by the risk for suicide.

**Figure 6:**
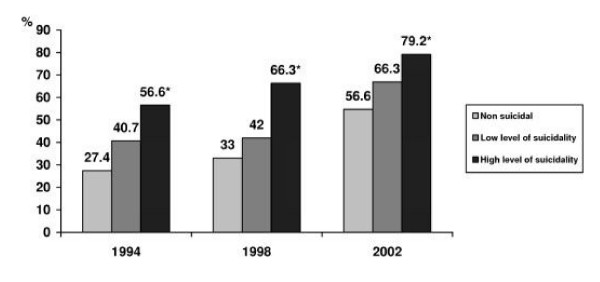
**The percentage of girls who approved a person's freedom to choose suicide, by level of suicidality in 1994, 1998 and 2002**. * – p < 0.05 by the risk for suicide.

It was observed that an approving attitude towards suicide among adolescents could be identified as a significant factor associated with suicidality. The Figures 5 and 6 show, correspondingly, the percentage of boys and girls in every level of suicidality who approved a person's freedom to choose suicide. This phenomenon is especially evident in 1994 and 1998 years of the survey, where the proportion of the adolescents expressing such an attitude among the individuals with high level of suicidality was approximately twice higher in comparison with those who did not express any suicidal ideation.

The suicidal risk impact of a positive attitude towards a person's freedom to choose suicide was measured by calculating the odds ratio (OR). The crude OR's of the low and high levels of suicidality in comparison to non suicidal, by gender, age and year of the survey, are given in the Table 3. Overall, a statistically significant suicidal risk impact of a positive attitude towards freedom to choose suicide was established in all the groups of the adolescents by gender, age and the study year. The observed associations were more evident among the girls.

**Table 3:** Crude odds ratios (OR) and 95% confidence intervals (CI) of low and high level of suicidality in relation to approving a person's freedom to choose suicide, by gender, age and year of the survey

Year of the survey	Gender and level of suicidality	11-year-olds		13-year-olds		15-year-olds		Total	
		OR	(95% CI)	OR	(95% CI)	OR	(95%CI)	OR	(95%CI)
	*Boys:*								
1994	Non suicidal	1		1		1		1	
	Low	1.48	(1.03–2.12)	1.77	(1.31–2.41)	1.86	(1.36–2.53)	1.27	(1.03–1.55)
	High	1.52	(0.91–2.55)	2.89	(1.70–4.89)	3.31	(1.78–6.15)	1.51	(1.09–2.08)
1998	Non suicidal	1		1		1		1	
	Low	1.34	(0.94–1.90)	1.75	(1.25–2.44)	2.08	(1.47–2.94)	1.45	(1.20–1.74)
	High	2.34	(1.37–4.00)	3.70	(2.09–6.53)	3.52	(1.74–7.10)	2.86	(2.06–3.96)
2002	Non suicidal	1		1		1		1	
	Low	1.64	(1.09–2.45)	1.63	(1.13–2.36)	1.63	(1.17–2.27)	1.63	(1.36–1.96)
	High	2.39	(1.02–5.57)	3.50	(1.75–7.00)	1.72	(0.95–3.12)	2.50	(1.74–3.59)
Total	Non suicidal	1		1		1		1	
	Low	1.27	(1.03–1.55)	1.45	(1.20–1.74)	1.63	(1.36–1.96)	1.47	(1.32–1.64)
	High	2.51	(1.09–2.08)	2.86	(2.06–3.96)	1.50	(1.34–3.59)	2.20	(1.82–2.67)

	*Girls:*								
1994	Non suicidal	1		1		1		1	
	Low	2.23	(1.62–3.07)	1.73	(1.32–2.28)	1.34	(1.02–1.77)	1.62	(1.34–1.95)
	High	5.40	(3.00–9.73)	2.24	(1.43–3.51)	3.15	(2.05–4.83)	3.33	(2.37–4.68)
1998	Non suicidal	1		1		1		1	
	Low	1.32	(0.92–1.89)	1.33	(0.98–1.82)	1.56	(1.14–2.15)	1.40	(1.19–1.65)
	High	2.79	(1.58–4.91)	3.63	(2.10–6.24)	4.54	(2.97–6.95)	2.81	(2.14–3.70)
2002	Non suicidal	1		1		1		1	
	Low	1.87	(1.31–2.70)	1.51	(1.12–2.04)	1.77	(1.32–2.38)	1.47	(1.24–1.73)
	High	3.15	(1.59–6.22)	3.32	(1.98–5.58)	3.13	(2.03–4.82)	3.51	(2.75–4.47)
Total	Non suicidal	1		1		1		1	
	Low	1.62	(1.34–1.95)	1.40	(1.19–1.65)	1.47	(1.24–1.73)	1.54	(1.39–1.69)
	High	3.33	(2.37–4.68)	2.81	(2.14–3.70)	3.51	(2.75–4.47)	3.40	(2.90–3.97)

We performed a multiple logistic regression analysis with the low and high level of suicidality versus having non suicidal as the dependent variables for gender, age and the year of the survey, and an attitude towards freedom to choose suicide as the independent variables (Table 4). Gender (girls vs boys), age (13- and 15-year-olds vs 11-year-olds) and attitude (approved vs not approved freedom to choose suicide) were the factors that increased the level of suicidality statistically significantly. Suicidal risk of the adolescents surveyed in the year 2002 was significantly reduced in comparison with their peers surveyed previously. The interaction terms among an attitude and gender, age and the year of the study were not significant with the exception of interaction between approval attitude towards suicide and gender for a high level of suicidality. This indicates a significant modification of interaction between positive attitude towards suicide and a high level of suicidality by gender group.

**Table 4:** Odds ratios (OR), 95% confidence intervals (CI) and significance levels (p) for gender, age, year of the survey and attitude towards suicide of the multiple logistic regression analysis

Variables in the equation	Dependent variable: *low level of suicidality *versus *non suicidal*			Dependent variable: *high level of suicidality *versus *non suicidal*		
	OR	(95% CI)	p	OR	(95% CI)	p
Girls vs boys (GIRLS)	1.71	(1.59–1.85)	<0.001	1.87	(1.65–2.12)	<0.001
13-year-olds vs 11-year-olds (AGE13)	1.82	(1.66–2.00)	<0.001	1.63	(1.40–1.91)	<0.001
15-year-olds vs 11-year-olds (AGE15)	2.50	(2.27–2.74)	<0.001	2.35	2.01–2.74)	<0.001
Survey in 1998 vs survey in 1994 (S1998)	1.00	(0.91–1.10)	0.98	1.16	(0.99–1.34)	0.058
Survey in 2002 vs survey in 1994 (S2002)	0.61	(0.56–0.67)	<0.001	0.73	(0.63–0.86)	<0.001
Approved vs not approved freedom to choose suicide (ATTITUDE)	1.63	(1.51–1.77)	<0.001	2.83	(2.49–3.23)	<0.001
ATTITUDE × GIRLS	1.03	(0.89–1.20)	0.69	1.51	(1.17–1.93)	0.001
ATTITUDE × AGE13	0.93	(0.77–1.12)	0.43	1.23	(0.90–1.68)	0.20
ATTITUDE × AGE15	0.98	(0.81–1.18)	0.81	1.28	(0.94–1.74)	0.12
ATTITUDE × 1998	0.90	(0.74–1.08)	0.24	1.24	(0.92–1.68)	0.15
ATTITUDE × 2002	0.95	(0.79–1.14)	0.59	1.00	(0.73–1.37)	0.98

## Discussion

The surveys of the recent years show that thoughts about suicide as well as suicidal behaviour are more characteristic to the young generation of nowadays than to the one some years ago. Lithuania, unfortunately, is not an exception. In the period of the last three decades the suicide rate among 15–18-year-olds increased much more dramatically than it has among general population [16]. Accidents, suicides and homicides make more than half of reasons in the mortality of the youngest inhabitants (less than 14 year old) [17]. This has led to an increased interest in suicidal threats and behaviour among adolescents.

Findings of the anonymous questionnaires conducted in different countries suggest that approximately 20%-30% of adolescents have been having thoughts of suicide and from 2% to 12% of young people claim about attempts to commit suicide [13,18-21]. On the other hand, population – based surveys designed to estimate the prevalence of suicidal ideation and behaviour, are often difficult to compare because of a great variability in definitions of suicidality and research methods used. Continuum of suicidality includes wide spectrum of suicidal thoughts and behaviour that begins with ephemeral thoughts about suicide and, proceeds to attempted suicide without injury, to attempted suicide with serious injury, and finally, to completed suicide. The cognitive development of children and adolescents also affects their understanding of the concepts of suicidal ideation and behaviour [22]. However, the data received in our study claim that suicidal indicators of Lithuanian adolescents remain constantly at a rather high level. According to the results of the 1998 survey, almost 40% of schoolchildren at the age of 11, 13, and 15 reported about suicidal thoughts, threats or attempts to commit suicide [23]. Relatively a low rate of reported suicide attempts, in comparison to the results of similar designed studies conducted in other countries may be influenced by young age and developmental state of respondents. Numerous researchers are prone to emphasize the developmental variations in death cognition. The concept of death develops in late childhood while full awareness of the implications of death is not gained until early adolescence [24].

The data of the year 2002 indicate that the frequency of schoolchildren's suicidal tendencies has slightly decreased. It is important to note, however, that these changes occurred due to the decrease in the group of the schoolchildren having "sometimes" suicidal thoughts. Another factor which influenced this change was significant decrease in the frequency of suicidality among the youngest boys. The decrease in the prevalence of suicidal tendencies among the 11 year-old boys could be somewhat attributed to an improved situation in schools, because in the period of the recent years more attention has been paid to the 11 year-olds' adaptation in schools (as it is a transitional period: children become pupils of a secondary school). A previous research of psychosocial correlates indicated a strong association *between *suicidal tendencies of 11-year-old boys with school difficulties [23]. On the other hand, the number of the schoolchildren with high suicidal risk indicators (often thoughts, concrete plans or attempts to commit suicide) has remained on the same high level. Such schoolchildren make up almost ten percent.

Analysis by gender revealed some changes in the patterns of the dynamics of boys and girls' suicidal tendencies during the period of eight years. According to the data of the first and the second survey, the frequency of the high level of suicidality among the boys was changing according to the age and had a tendency to decrease. The same indicators among the girls have an opposite trend and were increasing with age. Subsequently, the prevalence of a high level of suicidality was more characteristic for younger boys and elder girls. The last investigation, however, demonstrated similar rates to the ones found in most European countries: the numbers of adolescents contemplating suicide or making suicidal attempts increase correspondingly with age, girls' suicidal manifestations are almost twice higher than boys' [19,25]. This difference was not so evident among pupils aged eleven, but with every year of adolescence this difference increases due to the fact that the indicators the girls' suicidality changed faster than those of the boys.

The explanation of these patterns could not be homologous. Some authors associate this with different manifestations of adolescents' depression, pointing out the fact that girls with a medium level of depression tend to speak about suicide more often than boys [25]. According to other authors considering suicide as one of the possible alternatives when confronted with problems is rather a normal and prevalent way of coping in adolescents [26]. Nonetheless, the vast majority of the investigators tend to point out that any suicidal display, despite age and sex, must be treated seriously as a sign of possible suicide [27,28]. Most studies in samples of normal adolescents assume that suicidal ideation, threats and behaviour could be considered as a part in an overall continuum of suicidality [19,26]. A high prevalence rate of suicidal risk indicators among schoolchildren as well as the prognosis that a stable decrease of suicides is unexpected in Lithuania in the near future because of the rate of suicides in the young generation, born after 1970, make the situation urgent and stimulate a research in this field [16,23].

Culture influences timing, development, and shape of children's concept of death in general, and suicide in particular [29]. Despite a long standing research interest in the social correlates of suicide, the associations between attitudes and suicidal behaviour have been a topic of discussions and a growing research interest in the recent years. It has been marked, that suicide as a concept is more and more becoming a life norm and a suicidogenics atmosphere has widely set in our society [30,31].

The data of our survey demonstrated that children have increasingly expressed an acceptance of the choice to commit suicide. During the period of the last eight years the number of the adolescents supposing that a person has the right to decide – to live or to commit suicide – has credibly grown from 36,3% in the year 1994 to 62,5% in the year 2002. Consequently, an increasing number of the Lithuanian schoolchildren have been advocating suicide as a basic human right.

The most important finding of the present study is that an approving attitude towards suicide among adolescents is a significant predictor of suicidal ideation. The data of the surveys conducted in 1994, 1998 and 2002 confirmed a stable association between an approval attitude towards freedom to choose suicide and suicidal potential of the schoolchildren, aged 11, 13 and 15.

The adolescents who thought that suicide was an acceptable way – out of a complicated situation made an attempt of suicide twice more frequently than those who showed a negative attitude towards this phenomenon. Though gender differences were relatively small, boys were more likely than girls to endorse the idea that one has the right to take away one's life. The similar gender differences were observed in some other studies among adolescents [32]. Our results are consistent with the findings of the study of Israeli adolescents' attitudes to suicide that have suggested the participants' attitudes towards suicide correlate with their suicidal ideation. The more approving attitudes are being associated with a greater personal preoccupation with suicide [9]. The received data indicates that a tolerant view on suicide might be considered as a suicide risk factor. A positive attitude is working as a kind of a conduct influencing the action [30]. An approval attitude towards freedom to choose suicide in a complicated life situation could serve as a positive motivational force for death. Nowadays the rising suicide rates among youth may in part be attributable to that fact, that they are more tolerant of suicide and less fearful of its consequences.

A limitation of the present study is that the decision about student's attitude towards suicide was made referring to one question. Consequently, it may be possible to get a clearer picture of interaction between attitudes and person's suicidality using special attitude research scales. More the less, regarding high suicide rates in the society, a widespread attitude towards suicide as an appropriate choice among children and adolescents could be considered as very dangerous. These findings should be considered in the prevention of suicides. Norms, values and attitudes are not static. Usually these changes have taken place slowly and rather smoothly, but in order to affect suicidal risk of young people, such attitudes must be tackled purposefully.

## Conclusion

Based on the data provided by the current study it could be concluded that suicidal tendencies are quite frequent among schoolchildren; that consists a growing problem of public health in Lithuania. Moreover, the schoolchildren's attitude towards suicide became more agreeable: 36.6%, 41.9% and 62.5% of the respondents, correspondingly in 1994, 1998 and 2002, answered that they agree with a person's freedom to make a choice between life and suicide. Over the entire study period it was observed that an approving attitude towards suicide among the adolescents could be identified as a significant factor associated with suicidal ideation and suicidal behaviour. Therefore any attempt for development and implementation of a school program for prevention of suicides should consider these findings and set priorities accordingly.

## Competing interests

The author(s) declare that they have no competing interests.

## Authors' contributions

NZ outlined the questions on suicidal ideation and attitude towards suicide, substantially contributed to the conception and the design of the article and to the interpretation of data. AZ coordinated the surveys, performed the statistical analysis and helped to draft the manuscript. Both authors read and approved the final manuscript.

## Pre-publication history

The pre-publication history for this paper can be accessed here:


